# OP67 Advancing Toward Environmental Impact Assessment In Health Technology Assessment

**DOI:** 10.1017/S0266462325101244

**Published:** 2025-12-29

**Authors:** Mireille M. Goetghebeur, Rossella Di Bidino, Juliana Yi, Matthias Perleth, Rebecca Boyce, Li-Ying Huang, Fiona Campbell, Hans C. Ossebaard, Tara Schuller

## Abstract

**Introduction:**

The world is facing an urgent environmental emergency that calls for ambitious, coordinated action by governments to improve humankind’s relationship with nature. Mitigation (reducing pollution) and adaptation (adjusting to pollution) are both necessary, and “NextGen” health technology assessment can play a key role in helping to guide health system decision-makers to achieve environmental sustainability.

**Methods:**

In 2022, the International Network of Agencies for Health Technology Assessment (INAHTA) identified environmental impact assessment (EIA) as an important and urgent topic for a white paper. An international author group (n=10), formed of staff from INAHTA member agencies, wrote the paper. The author group met seven times over 2022 to 2024 to develop the paper, which is based on a literature review and expert opinion of INAHTA members. A second group of members formed an international advisory group (n=11), which reviewed the draft twice. The paper was approved by the INAHTA Board before release on the INAHTA website.

**Results:**

EIA can be useful to help achieve the “green” policy goals of health systems by identifying technologies that are comparatively harmful to the environment. However, health technology assessment (HTA) agencies are subject to the institutional structures and regulations of their local health system that can constrain their ability to independently adapt to incorporate EIA. Nevertheless, HTA agencies are starting to do this with some success, but difficult challenges remain, particularly around lack of data and methods. Looking ahead, raising awareness and working together across the HTA ecosystem will be key to achieving the shared goal of environmental sustainability.

**Conclusions:**

The successful inclusion of EIA in priority setting, assessment, and appraisal, as well as knowledge sharing and dissemination activities of HTA, is a challenge since the operating principles, methods, and data in this area are not yet mature. The successful inclusion of environmental impacts in HTA will require some reconsideration of existing value frameworks and methods.

